# Exercise Prescription Guidelines for Cardiovascular Disease Patients in the Absence of a Baseline Stress Test

**DOI:** 10.3390/jcdd7020015

**Published:** 2020-04-27

**Authors:** Megan Mytinger, Rachael K. Nelson, Micah Zuhl

**Affiliations:** School of Health Sciences, Central Michigan University, Mount Pleasant, MI 48859, USA; mytin1mb@cmich.edu (M.M.); nelso1rk@cmich.edu (R.K.N.)

**Keywords:** graded exercise testing, cardiac rehabilitation, aerobic exercise, cardiovascular disease

## Abstract

Aerobic exercise is a core component of cardiac rehabilitation (CR). Leading organizations recommend that the exercise prescriptions should be based on a symptom limited baseline graded exercise test (GXT). However, recent evidence suggests that only ~30% of CR clinics perform baseline GXTs. Consequently, exercise prescriptions including exercise progression in CR are not following standard exercise prescription guidelines. Therefore, the purpose of this review is to provide clinicians with evidence-based techniques for prescribing exercise in the absence of a baseline GXT. Intensity indicators (e.g., heart rate, perceived exertion) are reviewed, along with special exercise considerations for various disease states (e.g., heart failure, peripheral artery disease, and coronary artery disease). Baseline exercise testing remains the gold standard approach for prescribing exercise among heart disease patients, however, clinicians must be prepared to safely develop and monitor patients when a baseline GXT is not performed.

## 1. Introduction

Cardiac rehabilitation (CR) is a comprehensive treatment process that is centered on providing supervised aerobic exercise training, along with delivering ancillary services (e.g., nutrition counseling, smoking cessation, psychosocial support) for cardiovascular disease (CVD) patients. Prescribing safe and progressive exercise programs in CR is critical to improving functional capacity, which is a key prognostic indicator for CVD patients. Functional capacity is also termed maximal oxygen consumption (VO_2_max), or aerobic fitness, and is a measure of the hearts ability to deliver oxygen to tissue, and proficiency of the tissue to extract oxygen. It is commonly reported in metabolic equivalents (METs) where 1 MET is rest. The maximal MET value achieved during an exercise test can be used to determine VO_2_max (1 MET = 3.5 mL/kg/min), and workloads in cardiac rehabilitation are easily evaluated by calculating exercise METs. Evidence suggests that a 1 MET improvement in functional capacity after participation in CR confers a 17–30% decrease in all-cause mortality [1,2,3].

Leading organizations recommend that CVD patients should perform a symptom limited graded exercise test (GXT) prior to beginning their cardiac rehabilitation program [4]. The GXT provides accurate hemodynamic information that clinical exercise physiologists use to build patient specific exercise prescriptions. For example, precise peak heart rate and workload data allows exercise intensity to be based on a percentage of the peak values (e.g., %heart rate reserve or %VO_2_max). Additionally, a GXT can identify hemodynamic values and corresponding exercise workloads where symptoms (e.g., angina) or evidence (e.g., ST-segment depression) of myocardial ischemia occurs. This helps clinical exercise physiologists establish ischemic thresholds that should not be exceeded during exercise training in order to avoid eliciting ischemia. Despite this approach being the gold standard for exercise programming in CR [5,6], in a recent survey study among CR clinics in the United States it was reported that only 33% of clinics conduct baseline GXTs [7]. A similar survey of forty-five Dutch clinics identified that 36% of heart failure patients, and 30% of coronary artery disease patients did not perform a baseline GXT prior to beginning CR [8]. Possible reasons for not performing baseline GXT include, lack of reimbursement, patient safety concerns, inadequate equipment, and overall feasibility concerns. In response, many clinics perform sub-maximal testing such as the six-minute walk test. However, while these tests are helpful for estimating functional capacity, they are not used for exercise programming [9,10].

The absence of maximal baseline exercise testing raises an important discussion regarding exercise prescription techniques for CVD patients enrolled in CR. In particular, how do clinicians appropriately prescribe exercise to maximize improvements in functional capacity, but also maintain patient safety? Unfortunately, limited guidance is available for clinicians in this situation. Therefore, the primary purpose of this paper is to review available methods for prescribing exercise intensity and progressing patients in CR when a baseline GXT is not performed. Clinical considerations particular to heart failure, peripheral artery disease, and coronary artery disease patients will be highlighted. The practical application will assist clinicians in the field, and also educate students enrolled in clinical exercise degree programs.

## 2. Exercise Intensity Techniques

Ideally, peak heart rate and oxygen consumption values from a maximal effort GXT are used to set intensity for aerobic exercise prescription in CR. Commonly used indicators of intensity are percentages of heart rate reserve (%HRR), VO_2_ reserve (%VO_2_R), and peak aerobic power (%VO_2_max). Although large variations in exercise intensity targets for prescription exist among clinical organization and countries [11], the goal is to titrate exercise intensity to achieve 40–80% HRR, VO_2_max, or VO_2_R [11,12,13,14]. In addition, Borg’s rating of perceived exertion (RPE) 6–20 scale should be used to monitor the patient’s subjective intensity of exercise with a target of 12–16 (moderate to hard). When there is no data available from a baseline GXT, commonly used techniques for prescribing exercise include utilizing RPE and the resting heart rate plus 20–30 bpm method [12]. Recent research shows that subjective means for guiding exercise intensity, such as RPE, are closely related to physiologic responses to exercise, such as the lactate threshold, even in patients with coronary artery disease [15]. Although patients on beta-blocker therapy tend to report a higher RPE for a given exercise workload, an increase in RPE occurs as work rates increase and therefore remains consistent for prescribing exercise [16]. However, it is important to recognize that hemodynamic responses associated RPE values may vary among patients based on their disease pathology and severity, along with other medical therapies (e.g., pacemaker). Despite varying responses from diverse patient populations, the use of RPE guided exercise training has been successfully implemented into programs for a variety disease conditions [17,18]. It should be noted that the RPE scale must be accurately explained by clinicians, and understood by patients, to be used appropriately when prescribing exercise intensity.

Without a GXT, the first CR exercise session intensity goal is to target 20–30 bpm above resting heart rate (RHR +20–30) and monitor RPE to a range of 11–14 (somewhat hard to hard). Initial exercise workload should be adjusted up or down to reach these targets. If a patient is symptom free, and reports a low RPE (<11) at RHR +20–30, then exercise intensity may be increased so that the RPE goal of 11–14 is reached. Hemodynamics, exercise workload (treadmill speed and grade, or watts), as well as patient symptoms should be documented and used as a guide for progressing the exercise program. Exercise prescriptions using +20–30 bpm and RPE methods have been briefly studied among CR patients. Joo et al. compared exercise at 11–13 RPE (light to somewhat hard) and exercise at RHR +20 to gas exchange values from a maximal cardiopulmonary exercise test among 11 patients enrolled in CR [19]. Exercise at 11–13 RPE resulted in patients exercising at 71% VO_2_R, while exercise at RHR +20 equated to 42% VO_2_R. The RHR +20 method resulted in 55% of patients exercising between 40–60% VO_2_R for their first exercise session, falling within target goals for CR patients. However, the authors stated that the RPE 11–13 method led to patients exercising at an intensity level of vigorous (based on %VO_2_R) for their first exercise session, which may raise safety concerns [19]. Using a similar study design, Reed et al., 2017 compared RHR +20 or RHR +30 to %HRR during the first three weeks of CR in heart failure patients. Only 26% of the patients who were instructed to exercise at RHR +20, and 38% of the patients instructed to exercise at RHR +30 achieved 40–60% HRR, respectively [20]. Despite a low proportion of patients meeting exercise intensity guidelines, both groups demonstrated an increase in functional capacity (measured by 6 MWT) at the end of 12 weeks of cardiac rehabilitation training [20]. Collectively, these studies demonstrate that beginning exercise at RHR +20–30 will achieve target exercise intensity goals for some, but not all patients. Conversely, beginning RPE 11–13 or RPE 11–14 may result in patients exercising at a higher relative intensity during the first exercise bout. Therefore, it is even more important to closely monitor patient symptoms during the first exercise session while targeting exercise intensity goals when prescribing exercise intensity without a baseline GXT.

In addition, there are also several considerations that should be monitored to assist with establishing initial exercise workloads and for preserving safety during exercise training for various co-morbidities (e.g., hypertension, diabetes status) and lifestyle habits (e.g., physical activity behavior). Because exercise blood pressure is unknown in the absence of a GXT, blood pressure should be measured during exercise sessions to monitor possible hypertensive or hypotensive exercise responses. An exaggerated systolic and, or diastolic blood pressure response to exercise should be documented and aligned with relative exercise intensity as well as symptoms that may arise. More specifically, a SBP > 250 mmHg and, or DBP > 115 should be used as relative indications to terminate exercise. Also, a decrease in SBP > 10 mmHg (presently below baseline) that occurs with an increase in exercise workload should also be documented and evaluated for possible ischemia [4]. For diabetic patients, it is important to be cautious when prescribing higher initial exercise intensities in order to prevent any hypoglycemic events. Muscle contraction promotes skeletal muscle glucose that is directly proportional with exercise intensity [21]. Additionally, most diabetic patients are on one or more glucose lowering medications. Therefore, when exercise is coupled with anti-diabetes medications including insulin or medications that promote insulin secretion (e.g., GLP-1 receptor agonists) it can result in hypoglycemia. Therefore, blood glucose levels should be check before and after the first few exercise sessions for diabetic patients. If blood sugar levels are low (<70 mg/dL), patients should be given a small snack (~15 g of carbohydrate) and their blood sugar checked again 15 min later. This should be repeated until the patient’s blood sugar is >70 mg/dL. Conversely, some patients can be prescribed initially higher exercise intensities. For example, it should be recognized that patients with a recent history of being physically active will likely be able to begin at higher exercise workloads. Overall, it is important to carefully consider each patient’s medical history including: cardiovascular risk factor assessment, medical diagnoses, symptoms, medications, and psychosocial history when prescribing exercise. This is particularly important when the diagnostic, or prognostic utility of the GXT is not available. In summary, every patient’s unique case should be comprehensively evaluated when prescribing initial workloads in CR.

## 3. Exercise Progression Techniques

Evidence suggests that CVD patients achieve larger improvements in functional capacity when they exercise at higher exercise intensities throughout CR [22]. In addition, coronary artery disease patients, who exercise at or below 3.5 METs upon completion of CR were characterized as a high-risk group with 3-year cardiac event rates above 18% [23]. Furthermore, our group recently reported that an increase in treadmill exercise workloads between CR sessions 12 to 24 and 24 to 36 predicted functional capacity improvements of 0.50 to 0.80 METs, respectively in a diverse group of patients [24]. Collectively this suggests it is of critical importance that CVD patients are progressed toward achieving higher exercise workloads throughout their CR program. However, in the absence of a baseline GXT, prescribing exercise using percent heart rate or gas exchange values from a maximal cardiopulmonary exercise test are not possible because peak exercise values are unknown. Therefore, the use of RHR method, RPE, and close monitoring of exercise symptoms will establish an initial safe exercise workload and be used to progress patients through CR. One recommendation is to begin the patient with 5–10 min or more of aerobic exercise at an RPE of 11–14 (light to somewhat hard), and a target heart rate of RHR +20–30 [25]. As always, exercise workload (e.g., treadmill speed and grade), patient’s heart rate, and any symptoms should be documented. Next, duration of exercise should be increased by 1–5 min per session until duration goal is achieved while simultaneously holding RPE in the 11–14 range [26]. Finally, a gradual increase in RPE (>14) can be encouraged as tolerated [25]. Importantly, an increase in RPE should accompany higher exercise workloads (increase in speed/grade or watts) and heart rate. If a patient reports a higher RPE value from a previous session, but no change in heart rate or exercise workload is observed, then there are two potential courses of action. The clinician should immediately explore why the patient is reporting a higher RPE, which may be a result of excess fatigue, or other symptoms. Symptoms of angina, dyspnea, chronotropic incompetence, and abnormal ECG findings should be continually monitored and documented. If a patient does present symptoms during exercise, then the documented heart rate and workload upon which symptoms occur will establish exercise thresholds. Future exercise sessions should be at an intensity below the threshold. Conversely, if the patient is otherwise okay and hemodynamically stable, the clinician should encourage the patient to increase exercise intensity. In general, there should be documentation of exercise progression each week of cardiac rehabilitation [26]. Table 1 provides a case study example of a progressive exercise program. Note that only duration of exercise is increased per session with RPE held constant at 11–14. The initial heart rate is RHR +20–30 above rest, but this may increase per session based on achieving the RPE target. In this example, the patient will be exercising continuously for 25 min by exercise session 4.

While exercise starting points (HR +20–30 above rest, RPE 11–14, 5–10 min) for each patient may be similar when a baseline GXT is not available, the rate of progression will vary tremendously among patients. Patients who are symptom free, tolerate increases in exercise intensity, and are motivated will progress to high intensity exercise in a shorter time frame. Importantly, if risk of adverse events are properly assessed and unlikely, then clinicians are recommended to avoid undue restriction in progressing exercise intensity [26]. In support of this recommendation, exercise METs achieved in CR were reported to be inversely related to re-hospitalizations, nonfatal myocardial infarctions, and mortality [23]. This highlights the importance of continually progressing patients to higher exercise intensities throughout CR. For this reason, high intensity interval training (HIIT) exercise has gained popularity as a more effective exercise program than traditional continuous exercise for improving functional capacity among CVD patients [27,28]. This is particularly true for heart failure patients [29]. However, in a recent safety evaluation of HIIT, authors recommend that a baseline GXT with negative ECG result is of vital importance before beginning a HIIT program [30]. This assessment is logical because HIIT exercise prescriptions (e.g., 30 s to 4 min at 80–90% HRR) are commonly based on data extracted from the GXT such as peakHR [31,32]. Despite the recommendation from Wewege et al., 2018 there are no clear guidelines or data to suggest that patients without a baseline GXT should not participate in HIIT exercise. Therefore, the clinical exercise physiologist is faced with this decision on whether a baseline GXT is necessary for developing a HIIT prescription. This determination should be based on each individual patient’s stability of symptoms, exercise tolerance, and progression from previous supervised exercise sessions. In addition, co-morbidities, patient age, exercise history, and complexity of their cardiovascular disease state should be considered. In general, patients without a baseline GXT should begin CR with traditional continuous aerobic exercise for several weeks to months. If symptom free throughout previous supervised exercise sessions then the clinicians may decide to implement HIIT exercise.

## 4. Clinical Considerations

### 4.1. Chronic Heart Failure Patients

Chronic heart failure (CHF) affects nearly 26 million people globally, and data suggests prevalence is increasing substantially [33]. For example, in the US, there were 6.2 million cases of CHF reported between 2012–2016, and is expected to reach 8 million by 2030 (Benjamin et al., 2019). The disease is classified into heart failure with reduced ejection fraction (HFrEF), heart failure with preserved ejection fraction (HFpEF), and more recent, heart failure with mid-range ejection fraction (HFmEF). CHF is complex, and often presents with exercise intolerance characterized by shortness of breath (i.e., dyspnea) or fatigue with minimal exertion. The benefits of CR among CHF patients are demonstrated by evidence of 30% reduction in mortality and hospitalizations when adhering to properly prescribed exercise [13,34]. 

In the absence of a baseline GXT, it is generally recommended that CHF patients begin exercising at an RPE between 11–13 (light to moderate), and work to achieve a range between 12–14 (moderate to hard). Duration should be initially increased to achieve 30 min of continuous exercise followed by an upward adjustment in exercise intensity [13]. Exercise volume goals in CR are to complete 3–7 MET hours per week [13]. For example, if a patient exercises at 3 METs for 1.5 h per week then they would achieve 4.5 MET hours per week (3 METs × 1.5 h).

CHF patients are considered to be at the highest risk for an exercise-related event, and require attentive supervision [14]. However, exercise-induced arrhythmias, shortness of breath, as well as blood pressure and heart rate responses to exercise are unknown without a baseline GXT. Therefore, clinicians must be prudent in detailing notable symptoms, along with aligning any ECG and/or blood pressure abnormalities that occur as exercise intensity is progressed. If clinicians observe progressive worsening of exercise intolerance, or significant ischemia at low work rates (<2 METs) exercise should be terminated and the patient should be reassessed [35]. In addition, CHF patients commonly present with complex treatment interventions such as bi-ventricular pacemakers, implantable cardioversion devices (ICDs), along with medications that influence exercise responses such as anti-arrhythmia and inotropic agents [35]. These conditions do not preclude CHF patients from CR, but considerations should be made to maximize the patient’s ability to perform exercise safely. For example, heart rate during exercise must be kept below established thresholds for ICD activation, and limit settings or rate responsiveness of pacemakers should be known [35]. The clinical exercise physiologist must work closely with each CHF patient’s physician to optimize their CR exercise prescription.

### 4.2. Peripheral Artery Disease Patients

The global prevalence of peripheral artery disease (PAD) is estimated to be 200 million cases, and the US recently reported nearly 9 million PAD cases among adults over 40 years of age (Benjamin et al., 2019). PAD is associated with a nearly 2-fold increase in cardiovascular mortality in both men and women [36]. PAD is defined as partial or complete occlusion of arteries of the lower limbs, primarily due to atherosclerotic disease. Stenosis of the vessels reduces oxygen delivery to muscle tissue, and may cause leg pain during exertion that is relieved upon rest (i.e., intermittent claudication). Progressive, or untreated PAD results in critical limb ischemia, which presents as pain at rest, tissue damage, and possible limb amputation [36]. 

Among patients with PAD, aerobic exercise has profound benefits for improving walking distance, and pain free walking [37]. Despite an early meta-analysis (25 years ago), demonstrating that treadmill exercise improved pain free walking by 176% among PAD patients [38], it wasn’t until recently (2017) that the US Centers for Medicare and Medicaid Services approved 36 sessions of supervised exercise training for patients suffering from PAD symptoms of intermittent claudication [39]. This was a substantial and important step forward to enrolling PAD patients in CR programs. Yet this poses a new challenge for clinical exercise physiologist to appropriately prescribe exercise to maximize improvements not only in functional capacity but also walking distance and pain free walking for PAD patients. A symptom limited treadmill GXT is useful among PAD patients for evaluating functional capacity, and possible symptoms of CAD, especially because of the strong association between PAD and CAD [40]. PAD patients may terminate exercise early into a GXT because of moderate to severe claudication. It is imperative that a treadmill (rather than other exercise modality such as cycle ergometry) test is used to establish claudication onset time (walking speed when claudication occurs), and peak walking time (the time in which claudication forces patient to stop walking) [40]. In regard to exercise prescription in CR, it is recommended that PAD patients walk at the speed and grade where the onset of claudication occurs and continue walking until pain reaches moderate level (3 out of a 4-point scale). The patient should be encouraged to continue walking beyond the pain threshold for 5 to 10 min followed by a rest interval and then repeat (Gerhard-Herman, 2017). The goal is to achieve 30 min total of walking exercise comprised of several 5–10 min bouts [37]. The approach of walking past the pain threshold followed by recovery has been shown to extend claudication onset by 86% and walking distance covered by 87% [41].

If treadmill testing is not performed, then the onset of claudication should be documented during the patient’s first CR exercise session and used to set walking workloads for subsequent exercise bouts. The goal of completing 30 min of walking at moderate leg pain should remain. As the patient progresses, treadmill walking speed should be routinely increased with a goal of sustained walking at 3.0 mph [25,42]. Finally, asymptomatic PAD patients should be monitored under the same guidelines as other CVD patients enrolled in CR by using RHR +20–30 and RPE for their initial exercise session.

### 4.3. Coronary Artery Disease

The prevalence of coronary artery disease (CAD) in the US is estimated to be 18.2 million cases, and nearly 50% of all CVD-related deaths are due to CAD. Over 9 million deaths from CAD were reported globally in 2016, and it remains the leading cause of death in countries of all income groups [43,44]. CAD patients commonly present with symptoms of angina (i.e., chest pain) and shortness of breath (i.e., dyspnea), along with ST-segment displacement on an ECG during exercise. A GXT is important for establishing exercise intensity at which symptoms occur, and is reported as the ischemic or anginal threshold [45]. Exercise intensity during each CR session should be below the patient’s ischemic threshold. More specifically, exercise heart rate should be 10 bpm below the heart rate at which symptoms of angina, or ST-segment depression occurs [45]. In the absence of a GXT the initial exercise prescription should follow standard guidelines (RHR +20–30, RPE 11–14). Symptoms and ECG should be diligently monitored by the clinicians. If the patient reports mild angina or ST-segment depression is observed (≥1 mm from baseline) during a CR session then heart rate should be used to establish the ischemic threshold, and future exercise bouts should be 10 bpm below the identified threshold. Evidence suggests that ventilatory anaerobic threshold occurs prior to the ischemic threshold during exercise among CAD patients. This demonstrates that exercise below the ischemic threshold should still provide an adequate exercise stimulus to cause meaningful cardiovascular adaptations [46].

## 5. Conclusions

In the absence of a baseline GXT, exercise intensity guidelines for prescribing exercise among CVD patients in CR include beginning the patient at an exercise intensity equal to RHR +20–30 and RPE goal of 11–14. The initial duration is to achieve 10 continuous minutes of exercise and increase 1–5 min per session until a duration goal of 40–60 min is achieved. In regards to progression, duration should be achieved before intensity is progressed. Intensity should then be systematically increased by monitoring RPE until the patient is consistently exercising at an RPE of 12–16 (moderate to hard). The patient should be closely monitored early on in CR to observe any exercise-induced symptoms (e.g., angina, claudication). Although these guidelines appear vague, they may provide a safe and effective starting point for most CR patients. Ultimately progression of exercise should be relative to each patient’s risk stratification, underlying disease state, exercise tolerance, and goals. However, it is vital that stable patients are continually progressed throughout CR, and do not remain at the same workloads for consecutive time frames (weeks or months). This is a potential pitfall that can occur if a patient consistently reports RPE values between 12–16 in the absence of increased workload. In addition, the RPE and RHR approaches can be used to monitor and guide patients who are candidates for home-based cardiac rehabilitation.

## 6. Future Directions

Unfortunately, limited empirical data exists comparing functional outcomes in response to exercise prescription techniques in CR when a baseline GXT is not used to set exercise intensity. In addition, there has been no established HIIT protocols for CR patients when baseline GXT data (peak heart rate) is unknown. Research in these two areas are of critical importance for clinicians because the majority of CR clinics do not perform baseline GXTs [7].

## Figures and Tables

**Table 1:** Example of a progressive exercise program in cardiac rehabilitation.

Exercise Session	Duration	RPE (6–20)	Heart Rate	Mode	Comment
1	10 min	11–14	Target: 92–102 bpm	Treadmill: begin patient at 1.5–2.5 mph	Record treadmill workload, heart rate achieved, and symptoms
2	15 min	11–14	Target: heart rate may need to be increased to meet RPE goal	Treadmill: increase walking speed or grade if appropriate. Cycle/recumbent: introduce new modality	Record exercise workloads, heart rate achieved, and symptoms.
3	20 min	11–14	Target: heart rate may need to be increased to meet RPE goal	Treadmill: increase walking speed or grade if appropriate. Cycle/recumbent: introduce new modality	Record exercise workloads, heart rate achieved, and symptoms
4	25 min	11–14	Target: heart rate may need to be increased to meet RPE goal	Treadmill: increase walking speed or grade if appropriate. Continue to introduce new exercise modes if appropriate	Record exercise workloads, heart rate achieved, and symptoms

Case study example: female patient, age 65, resting HR = 72 bpm, diagnosed CAD with PCI and coronary stent treatment 14 days ago. Min = minutes, RPE = rating of perceived exertion.
